# Rapid Detection of Volatile Organic Metabolites in Urine by High-Pressure Photoionization Mass Spectrometry for Breast Cancer Screening: A Pilot Study

**DOI:** 10.3390/metabo13070870

**Published:** 2023-07-21

**Authors:** Ming Yang, Jichun Jiang, Lei Hua, Dandan Jiang, Yadong Wang, Depeng Li, Ruoyu Wang, Xiaohui Zhang, Haiyang Li

**Affiliations:** 1 Key Laboratory of Separation Science for Analytical Chemistry, Dalian Institute of Chemical Physics, Chinese Academy of Sciences, Dalian 116023, China; yangm@dicp.ac.cn (M.Y.); jjc@dicp.ac.cn (J.J.); lhua@dicp.ac.cn (L.H.); jiangdandan@dicp.ac.cn (D.J.); 2 College of Environment and Chemical Engineering, Dalian University, Dalian 116000, China; lidepeng@dlu.edu.cn; 3 Center for Advanced Mass Spectrometry, Dalian Institute of Chemical Physics, Chinese Academy of Sciences, Dalian 116023, China; 4 Department of Oncology Medicine, Affiliated Zhongshan Hospital of Dalian University, Dalian 116023, China; wangruoyu1963@163.com (R.W.); wangyadong@dlu.edu.cn (Y.W.)

**Keywords:** high-pressure photoionization mass spectrometry, urine, volatile organic metabolites, breast cancer, rapid detection

## Abstract

Despite surpassing lung cancer as the most frequently diagnosed cancer, female breast cancer (BC) still lacks rapid detection methods for screening that can be implemented on a large scale in practical clinical settings. However, urine is a readily available biofluid obtained non-invasively and contains numerous volatile organic metabolites (VOMs) that offer valuable metabolic information concerning the onset and progression of diseases. In this work, a rapid method for analysis of VOMs in urine by using high-pressure photon ionization time-of-flight mass spectrometry (HPPI-TOFMS) coupled with dynamic purge injection. A simple pretreatment process of urine samples by adding acid and salt was employed for efficient VOM sampling, and the numbers of metabolites increased and the detection sensitivity was improved after the acid (HCl) and salt (NaCl) addition. The established mass spectrometry detection method was applied to analyze a set of training samples collected from a local hospital, including 24 breast cancer patients and 27 healthy controls. Statistical analysis techniques such as principal component analysis, partial least squares discriminant analysis, and the Mann–Whitney U test were used, and nine VOMs were identified as differential metabolites. Finally, acrolein, 2-pentanone, and methyl allyl sulfide were selected to build a metabolite combination model for distinguishing breast cancer patients from the healthy group, and the achieved sensitivity and specificity were 92.6% and 91.7%, respectively, according to the receiver operating characteristic curve analysis. The results demonstrate that this technology has potential to become a rapid screening tool for breast cancer, with significant room for further development.

## 1. Introduction

The global prevalence of female breast cancer (BC) has surged to 11.7%, accounting for approximately 2.3 million cases, thus surpassing lung cancer as the most frequently diagnosed malignancy. Additionally, it stands as the fifth major contributor to cancer-related fatalities worldwide, claiming the lives of 685,000 individuals [1,2]. The incidence and mortality rates of breast cancer exhibit an upward trend. Prior research indicates that the fatality rate associated with breast cancer could be significantly reduced through timely detection and comprehensive treatment [1,2,3]. Presently, mammography serves as the conventional modality for breast screening; however, it exhibits diminished sensitivity towards detecting small tumors, is constrained by patient age limitations, and cannot yield definitive disease outcomes [4,5]. Furthermore, ultrasound and magnetic resonance imaging (MRI) are commonly employed in conjunction with mammography to identify minute lesions that may evade detection through mammography alone. However, these methods exhibit relatively lower specificity, and their costly nature can potentially contribute to instances of overdiagnosis [6,7,8]. Therefore, there exists a pressing demand for novel operational strategies that can be readily implemented on a wide scale within practical clinical settings for breast cancer screening.

Over the past few decades, a multitude of platforms leveraging omics technology have been developed and extensively employed in the realm of disease diagnosis and screening, encompassing not only cancer but also its distinct subtypes. Numerous molecular constituents, such as genes, proteins, and metabolites, have been proposed as potential biomarkers for breast cancer [9,10,11,12]. Metabolomics represents a robust and auspicious avenue for examining the intricate interplay between metabolites and physiopathological alterations through comprehensive qualitative and quantitative analysis of all organismic metabolites [10,12,13,14]. This approach harbors immense potential to discern and identify heterogeneous tumor diseases during their nascent stages [9]. Urine, serving as an optimal biofluid for metabolomic investigations, boasts several advantages, including non-invasive sampling, easy accessibility, and lower protein content, thereby reducing complexity. In addition, compounds produced by the body’s metabolism need to be concentrated by the kidneys before being excreted, making urine a rich source of metabolites [15]. Numerous volatile organic metabolites present in urine offer abundant insights into the onset and progression of diseases. Previous research has demonstrated that tissues generate distinct VOMs or exhibit altered concentrations of VOMs in pathological states, encompassing infections, neoplasms, and metabolic disorders [15,16,17].

To detect VOMs in urine, certain analytical techniques based on gas chromatography-mass spectrometry (GC-MS) have been utilized by integrating static/dynamic head-space-solid phase microextraction or stir bar extraction methodologies [18]. Some potential biomarkers for cancers, such as lung cancer [13], prostate cancer [19], breast cancer [4], and gastric cancer [20] have been successfully identified. Nevertheless, the GC-MS methods necessitate intricate pretreatment procedures and prolonged analysis durations, rendering them unsuitable for high-throughput and large-scale disease screening. Direct mass spectrometry based on soft ionization techniques, such as proton transfer reaction mass spectrometry (PTR-MS), selected ion flow tube mass spectrometry (SIFT-MS), and photoionization mass spectrometry (PI-MS) has been successfully used for rapid detection of trace volatile organic compounds in a complex matrix. Huang et al. used SIFT-MS to analyze urine headspace of gastro esophageal cancer patients and found seven statistically different VOMs [14]. PTR-MS was used in gastric cancer patients for VOM analysis in breath gas by Yoon et al. [21]. A high-pressure photoionization time-of-flight mass spectrometry (HPPI-TOFMS) has recently been developed with the advantages of high sensitivity, fast response, and good moisture resistance, which is especially suitable for rapid detection of trace volatiles and has been widely used in other fields [22,23,24,25]. It has shown excellent performance in the detection of exhaled breath, with the limits of detection (LODs) as low as 0.015 ppb for aliphatic and aromatic hydrocarbons [23], and has been successfully applied in the early screening of lung and gastroesophageal cancers [26,27]. HPPI-TOFMS has also been successfully used in the detection of VOMs in human urine with the LOD for trimethylamine as low as 100 ng L^−1^ under alkaline conditions, and a new biomarker 2,5-dimethylpyrrole was exclusively found in the smoker’s urine sample in addition to toluene [24].

In this study, the integration of HPPI-TOFMS with the dynamic purge-injection method was employed for the rapid and highly sensitive detection of volatile compounds in urine. A straightforward pretreatment approach involving the addition of acid and salt was implemented and investigated for VOM sampling from urine samples. After optimizing the experimental conditions, the method was applied to analyze urine samples obtained from 24 breast cancer patients and 27 healthy controls. The resulting MS data were subjected to statistical analysis to identify distinctive VOMs in urine samples between breast cancer patients and the healthy control group. Subsequently, the model’s classification efficacy was assessed by constructing a receiver operating characteristic (ROC) curve.

## 2. Materials and Methods

### 2.1. Instrumentation

**Figure 1:**
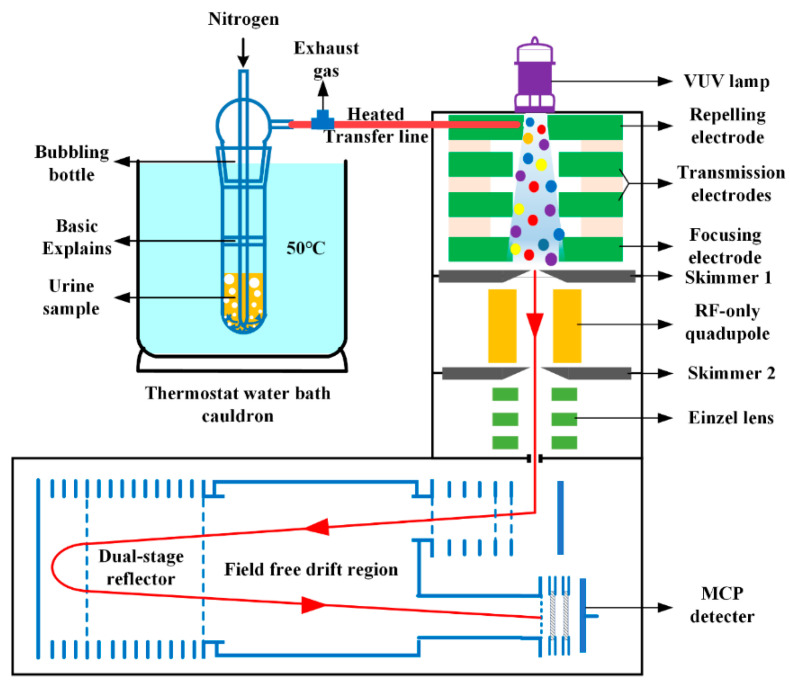
Schematic diagram of the HPPI-TOFMS system combined with dynamic purge-injection apparatus.

The home-built HPPI-TOFMS was composed of a HPPI ion source, an ion transmission system, and an orthogonal acceleration reflectron mass analyzer (see Supporting Information, S1). As shown in Figure 1, the HPPI ion source consisted of a vacuum ultraviolet krypton (VUV-Kr) lamp (Heraeus Noblelight Ltd., Shenyang, China) and a high-pressure photoionization region, which was constructed by five annular stainless steel electrodes: a repelling electrode (6 mm i.d., 5 mm thick), two identical transmission electrodes (14 mm i.d., 5 mm thick), a focusing electrode (14 mm i.d., 5 mm thick), and a Skimmer-1 electrode (1 mm i.d., 4 mm thick). Three 1 mm thick polyether-ether-ketone (PEEK) insulation annular washers (16 mm i.d.) were employed to separate the electrodes, except for the space between the last focusing electrode and Skimmer-1 electrode for an excess neutral exhaust. All the electrodes were electrically connected by using a 1 MΩ resistor string, and additionally, the Skimmer-1 electrode was further connected by another 1 MΩ resistor to the ground. The voltages applied on the repelling electrode and Skimmer-1 electrode were 18 V and 12 V, respectively, while a voltage of 16 V was applied on the focusing electrode to form a nonuniform electric field in the ionization region, which was utilized for ion focusing and higher ion transmission efficiency. A mass resolving power of 5000 (full width at half-maximum, FWHM) was achieved with a 0.5 m field-free drift tube. All the mass spectra were accumulated for 10 s at a repetition rate of 25 kHz, and all data were obtained by averaging results from six parallel measurements.

A dynamic purge-injection apparatus, composed of a thermostat water bath cauldron and a bubbling bottle with 20 mL inner volume, was employed for VOM sampling from urine samples into gaseous phase, as shown in Figure 1. The structure of the bubbling bottle was basically the same as that in our previous work [24], except for the addition of a porous glass cushion in the middle of the bottle, which was used to prevent the foam generated by bubbling from entering the sampling tube. A heated transfer line, containing a stainless steel capillary, 250 μm i.d. and 50 cm length, was used as the sampling tube to directly introduce gaseous VOMs from the outlet of the bubbling bottle into the ion source.

### 2.2. Chemicals and Reagents

Concentrated hydrochloric acid (AR, 36~38%) was purchased from Xilong Scientific Co., Ltd. (Guangdong, China). Sodium chloride (GR, 99.8%) was purchased from Shanghai Aladdin Bio-Chem Technology Co., Ltd. (Shanghai, China). Purified water was purchased from Hangzhou Wahaha Group Co., Ltd. (Hangzhou, China). Hydrochloric acid solution (4 mol·L^−1^) was prepared by diluting concentrated hydrochloric acid with purified water. High-purity nitrogen gas (99.999%) was provided by Dalian Institute of Chemical Physics, Chinese Academy of Sciences (Liaoning, China) and used as the gas source for the dynamic purge system. 

### 2.3. Urine Sample Collection, Preparation, and Detection

The middle stream of morning urine samples was collected from 24 breast cancer patients (BC, age 42–76 years, mean 52) and 27 healthy controls (CTL, age 18–61 years, mean 44) at Affiliated Zhongshan Hospital of Dalian University. All the urine samples were frozen at −80 °C immediately after sampling and thawed at 4 °C before detection. The study protocol was approved by the local ethics committee of Affiliated Zhongshan Hospital of Dalian University, and the method was carried out according to the approved guideline (2022021). Informed consent was obtained from all participants.

The urine samples were analyzed in four different conditions: (1) pure urine; (2) salted condition with addition of 1.0 g NaCl in 4 mL of pure urine; (3) acid condition with addition of 100 µL HCl (4 mol·L^−1^) in 4 mL of pure urine to adjust pH at 1; and (4) acid–salted condition with addition of 100 µL of HCl (4 mol·L^−1^) and 1.0 g NaCl in 4 mL of pure urine to adjust pH at 1. These samples were well mixed under ice and water bath conditions, stored at 4 °C and tested within 24 h. A urine pool noted as quality control (QC) was prepared by mixing the urine specimens (each with a volume of 400 μL) of all the participants in this study. The QC sample was processed in the same conditions and detected on every ten samples.

For VOM analysis, 4 mL of each urine sample was loaded into the clean bubbling bottle, which was sealed in 50 °C water bath. Subsequently, a high-purity nitrogen stream with 100 mL·min^−1^ was purged into the urine sample and produced a large number of small bubbles. Large quantities of VOMs were released into the gaseous phase by bubbles bursting and taken into the HPPI source through the stainless steel capillary for MS analysis. As the sampling flow rate of the inlet capillary was 50 mL·min^−1^, the extra gas was exhausted by a stainless steel tee connected before the capillary. The heated transfer line and ionization region were maintained at 100 °C throughout the whole analysis process to prevent condensation of the VOMs. Data acquisition of each mass spectrum was started from the introduction of purge gas and accumulated for 2 min. The entire experimental process, from the start of sample preparation to the end of data acquisition, took only about 4 min.

### 2.4. Statistical Analysis

The data were divided into two groups, i.e., BC group and CTL group. All the data points with signal intensity values below 20 counts were set to 0 to avoid interference from the background noise. Variables with non-zero values of intensity in at least 90% of each group were included in the data set; otherwise, the variables were removed. Afterwards, data filtering and normalization were performed to obtain a two-dimensional matrix containing metabolite information (the data can be found in the Excel file named “DATA” provided in the supporting materials). Multivariable analyses were carried out using SIMCA-P software (version 14.0, Umetrics, Umea, Sweden) with unit variance scaling (UV scaling). The principal component analysis (PCA) and partial least squares discriminant analysis (PLS-DA) models were built among different groups. The Mann-Whitney U test was used for the nonparametric test and implemented by Multi Experiment Viewer (MeV, version 4.9.0, TIGR, Boston, MA, USA). Mass peaks with variable importance of the projection (VIP) > 1 and *p*-value < 0.05 were selected and used to determine the statistically significant VOMs. Binary logistic regression analysis and ROC analysis of combinational VOMs were figured out by using PASW Statistics 25 software (SPSS, Chicago, IL, USA). Ten-fold cross validation was performed by an online metabolomics data analysis website MetaboAnalyst 5.0 to test the discrimination power of the combination of statistically significant VOMs.

## 3. Results

### 3.1. Influence of Acid and Salt Addition

**Figure 2:**
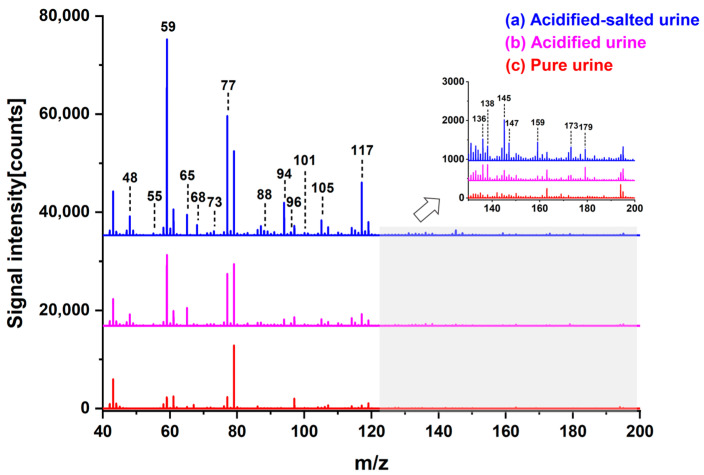
The comparison of mass spectra for different treatment methods of urine.

Acidification and alkalization of urine are prevalent pretreatment methodologies utilized for the extraction of VOMs during urine sampling. In our previous work, the VOMs identified in alkalized urine predominantly consisted of nitrogen-containing alkaline compounds, including dimethylamine, trimethylamine, piperidine, and dimethyl pyrazine [24], which were absent in the potential biomarker list from previous works by others [28,29]. Therefore, the pretreatment method for acidification (HCl) of urine was employed and investigated in this work. Adding acid can lower the pH of urine, which enhances the volatilization of acidic compounds, such as carboxylic acids, aldehydes, ketones, alcohols, etc., from the urine into the headspace, thus improving the detection sensitivity of these compounds [4,30]. In addition, NaCl was added in the urine sample to promote the volatilization of VOMs in urine, as the solubility of VOMs would decrease when the concentration of salt increased in the solution, known as the “salting-out effect” [31]. The addition of salt modifies the matrix of the sample by increasing ion activity. A significant quantity of the water molecules will exist as hydration associated with the ions in the solution under a high concentration of salt. VOMs do not dissolve well in the solution, which is bonded to the ions. Therefore, the solubility of VOMs in the liquid phase will decrease, and more VOMs move into the gas phase [31]. A mixed urine sample from four healthy volunteers (each with a volume of 20 mL) was used to evaluate the influence of HCl and NaCl addition. The signal intensities of over 33 mass peaks increased by more than 2-fold, and the signal enhancement of mass peaks with *m*/*z* 48, 59, 65, 77, and 94 even reached 11- to 21-fold after acidification of the mixed urine, as shown in Figure 2. Furthermore, 19 new peaks appeared in the acidified urine. After adding salt into the acidified urine, the signal intensity of mass peaks was further enhanced up to 62-fold (*m*/*z* = 94), compared with the pure mixed urine. Finally, based on putative annotation (level 2) [32], the measured masses of the characteristic ions were compared with their theoretical masses with a mass error of less than 30 ppm, resulting in the identification of several compounds as shown in Table 1. 

### 3.2. Multivariate Statistical Analysis

**Figure 3:**
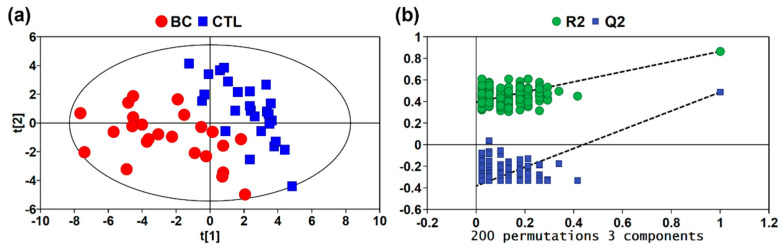
Multivariable analysis: (**a**) PLS-DA score plot (R2Y = 0.864, Q2 = 0.487). (**b**) Cross-validation plot of PLS-DA analysis, with a permutation test repeated 200 times and intercepts: R2 = (0.0, 0.394) and Q2 = (0.0, −0.383).

The processed MS data of BC and CTL groups were imported into SIMCA-P for PCA and PLS-DA analysis. During the urine sample analysis of BC and CTL, a QC detection was inserted for every ten samples. Five QC mass spectra were obtained, and clustered tightly together on the score plot of the PCA (see the Supporting Information, Figure S2a). Furthermore, the relative standard deviations (RSDs) of about 94% of the mass peaks were less than 30% for the QC sample (see the Supporting Information, Figure S2b), which exhibited the satisfactory repeatability and reliability of the method. PLS-DA maximizes the differences between samples by utilizing the biological measurements or category information in the Y-matrix, which could effectively solve the classification problem of metabolic phenotypes. As shown in Figure 3a, the BC group could be well separated from the CTL group from the score plot of PLS-DA, which indicated that the metabolite profiles could be well distinguished between the two groups. The cross validation with 200 iterations was performed, and the result shown in Figure 3b indicated that the PLS-DA model was not overfitted as the R2- and Q2-intercept values were 0.394 and −0.383, respectively.

### 3.3. Differential Metabolites in Urine of BC Patients

Univariate analysis was performed on the Multi Experiment Viewer, and the Mann-Whitney U test was used here to assess the significance of the selected candidate metabolites. Generally, a *p*-value < 0.05 was considered significant for the selected metabolite with a statistical significance. Furthermore, the variable importance for the projection (VIP) was plotted to summarize the importance of MS peaks, and only VIP > 1 can be reserved in the end. To further narrow down the range of significant candidate metabolites, the false discovery rate (FDR), based on the Benjamini–Hochberg correction, was introduced as another criterion. Metabolites that ultimately met a VIP > 1 and a *p*-value < 0.05 were selected as the differential metabolites. Finally, nine VOMs were identified as differential metabolites in the urine samples between BC patients and the CTL group, which could be classified as unsaturated aldehydes, ketones, aromatic hydrocarbons, volatile sulfur compounds, and heterocyclic compounds, as shown in Table 2.

**Figure 4:**
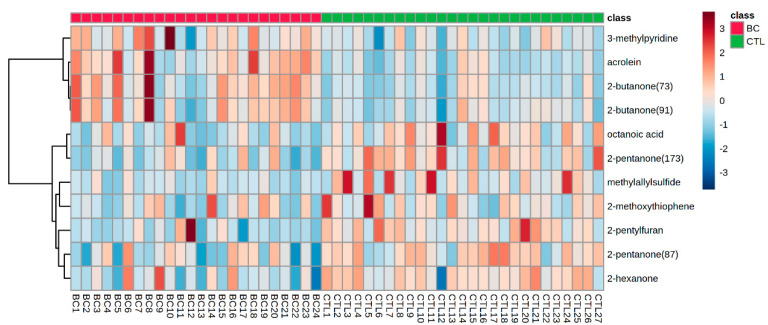
Heat map of the Pearson correlation coefficients between the differential metabolite contents.

Furthermore, hierarchical cluster analysis (HCA) was performed to better demonstrate the differences at metabolic levels between BC patients and the CTL group. The alteration of these VOMs in the urine of BC patients and the CTL group can be clearly observed in the heatmap as shown in Figure 4. The urine of BC patients had increased amounts of 2-butanone, 3-methylpyridine, and acrolein, but reduced concentrations of 2-pentyfuran, methyl allyl sulfide, 2-pentanone, 2-hexanone, octanoic acid, and 2-methoxythiophene. 

### 3.4. Receiver Operating Characteristic Curve Analysis

**Figure 5:**
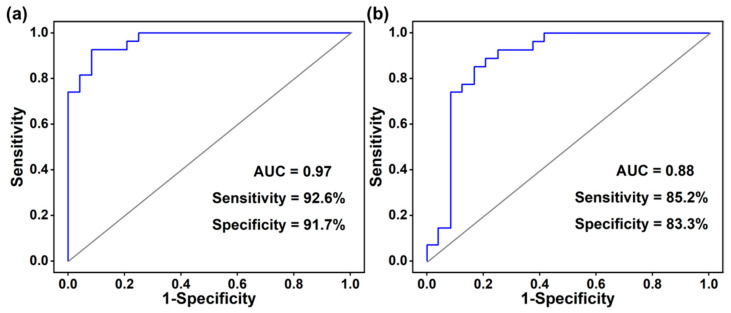
Receiver operating characteristic curve: (**a**) ROC curve of potential metabolic marker combination; (**b**) ROC curve of potential metabolic marker combination based on ten-fold cross validation.

The receiver operating characteristic curve is often used to evaluate the classification effectiveness of the model. However, the specificity and sensitivity of models containing a single differential metabolite for distinguishing BC patients from healthy controls were not definitive (see the Supporting Information, Table S1). A feasible solution for this problem is to combine more differential metabolites into a group for higher specificity and sensitivity. Therefore, the binary logistic regression analysis was employed to screen the differential metabolites to obtain an optimal metabolite combination. Eventually, three statistically significant metabolites, including acrolein, 2-pentanone, and methyl allyl sulfide were selected to build a metabolite combination model. This combination of metabolites has not been reported previously. The area under the ROC curve (AUC) of the statistically significant metabolic combination in the discovery set was 0.97, and the sensitivity and specificity were 92.6% and 91.7%, respectively, as shown in Figure 5a. The result indicated that this model has a good ability to identify BC patients. Subsequently, 10-fold cross-validation was performed to evaluate the model, as shown in Figure 5b, with the AUC = 0.88, sensitivity = 85.2%, and specificity = 83.3%, respectively. The results demonstrated the robustness of the model, which has the potential to be a useful tool for early screening of breast cancer.

## 4. Discussion

### 4.1. Potential Metabolic Pathway Analysis

The metabolic pathways of VOMs are pretty complex. As shown in Figure 4, the concentration of these VOMs were different between the BC and CTL groups, which is probably related to the increased oxidative stress and decreased apoptosis of cancer patients [14]. The relationship between the VOMs and cancer metabolism was not fully understood until now. The potential metabolic pathway of the five classes of the identified differential metabolites in Table 2 were summarized here according to previous studies.

Ketones are very abundant in urine. As shown in Table 2, there are three ketone compounds identified between the BC and CTL groups in this study: 2-butanone, 2-pentanone, and 2-hexanone. Different studies have shown that the ketogenic pathway may be directly related to tumor growth, and some ketones have been assigned as designated biomarkers for different cancers. Two potential pathways could be involved in their production: (i) oxidation of secondary alcohols catalyzed by ADHs (or cytochrome p450 (CYP2E1), and (ii) β-oxidation of fatty acids [20]. Therefore, 2-butanone, 2-pentanone, and 2-hexanone may be derived from 2-butanol, 2-pentanol, and 2-hexanol, respectively. But the source of these secondary alcohols remains unclear. They might stem from the oxidation of n-alkanes catalyzed by cytochrome p450 enzymes, microbial metabolism, or diet. Among them, 2-butanone and 2-pentanone have been detected as potential biomarkers in the breath gas of patients with gastric and ovarian cancers [34,35].

Although, only methyl allyl sulfide was identified as a differential sulfide compound, as listed in Table 2, sulfide compounds are generated by the incomplete metabolism of methionine and cysteine through the transamination pathway with high expression in urine [38]. On the one hand, during the transamination cascade, the methyl mercaptan produced by the conversion of methionine and cysteine is easily oxidized to produce a variety of volatile sulfides [38,39]. On the other hand, gram-negative bacteria can also produce these sulfur metabolites [40].

Additionally, there are volatile aldehydes in Table 2, which are common products of lipid peroxidation [30]. Acrolein is produced from the oxidation of arachidonic, linolenic, and linoleic acids in the presence of hydrogen peroxide and Fe^2+^ [35]. In addition to oxidative stress on unsaturated lipids, spermine and spermidine are potential carbon sources for acrolein. These compounds are oxidized by amine oxidase to corresponding amino aldehydes and spontaneously form acrolein in situ [33].

2-Pentylfuran was identified as the differential furan compound between the BC and CTL groups. Furans can be found in different exogenous sources, such as various foods. Furans are considered to be potential carcinogens, and high concentrations of furans can increase the probability of bile duct tumors in rats [41]. Additionally, furans have also been reported to be involved in anti-cancer defense mechanisms [42]. 2-Pentylfuran was found in the breath of patients with aspergillus fumigatus infections and human skin emanation [43]. Its production by natural dehydration of monosaccharides and oxidation of some fatty acids catalyzed by lipoxygenases could take place in adipocytes in the context of lipid peroxidation [43].

The last two differential metabolites in Table 2 are heterocyclic compounds, 2-methoxythiophene and 3-methylpyridine were detected in several reports and can even be considered as metabolic markers [4,20,30]. In Silva’s report, the concentration of 2-pentylfuran in BC patients is significantly higher than that in normal people, and it is considered as a biomarker of BC [4].

### 4.2. Methods Comparison and Limitations

GC-MS has become a core technology in metabolomic analysis due to its satisfactory performance in sensitivity and specificity [44]. Many researchers have utilized this technique to discover biomarkers for breast cancer in urine, achieving promising results [3,4,37,45,46,47]. Nevertheless, sample preparation is complex and time-consuming, involving multiple steps that restrict its application in high-throughput analysis and rapid screening. PTR-MS, as a highly sensitive direct MS technique, has also been applied to the detection of VOMs in urine [48,49]. However, the vast amount of water vapor from urine samples makes the ionization process more complicated and increases the difficulty of data. 

Compared to other methods, HPPI-TOFMS is more suitable for high-throughput urine sample analysis. Firstly, HPPI-TOFMS offers fast analysis speed and requires simple sample treatment steps such as acidification and salting. There is no enrichment or desorption process, and samples are directly detected after gasification. Secondly, a HPPI ionization source is less affected by humidity, enabling effective ionization of different compound types. As a soft ionization source, it avoids excessive fragmentation ions, making spectrum interpretation simpler. Thirdly, the instrument is easy to operate and has low maintenance costs. However, one drawback of HPPI-TOFMS is its reliance on high-resolution TOFMS for accurate qualitative analysis. Additionally, due to the lack of GC, it is unable to differentiate structural isomers.

Achieving positive results in a pilot study is encouraging; however, there are also some limitations of this study that need to be further addressed. The small sample size and lack of external validation in this study may limit the generalizability of the findings. Increasing the sample size would enhance statistical power and confidence in the results. External validation should be included to improve the reliability of the findings.

Additionally, confounding factors such as diet, medication, lifestyle, and clinical variables may influence metabolomic characteristics and introduce bias. Future research should employ appropriate methods to control for these factors and improve the reliability of the conclusions. Further research is needed to confirm the metabolic pathways and mechanisms underlying the associations between specific VOMs and breast cancer risk. In vitro and in vivo experiments are necessary to establish causal relationships and understand the biological significance of these findings.

## 5. Conclusions

This pilot study showcases a robust method for high-throughput analysis of VOMs in urine using the integration of high-pressure photoionization time-of-flight mass spectrometry with dynamic purge-injection. Its preliminary application in rapid breast cancer screening is demonstrated. VOMs present in urine samples are effectively volatilized and introduced into the HPPI-TOFMS system through dynamic purge-injection following the simple addition of acid and salt to the samples. The obtained mass spectrometry data were analyzed using partial least squares discriminant analysis and the Mann-Whitney U test, resulting in the identification of nine differential metabolites in the urine samples of 24 breast cancer patients and 27 healthy controls. Furthermore, a metabolite combination model was constructed using acrolein, 2-pentylfuran, and methyl allyl sulfide, which exhibited a satisfactory discriminatory performance (sensitivity = 92.6%, specificity = 91.2%) in distinguishing between breast cancer patients and healthy controls. Currently, the combination of HPPI-TOFMS with dynamic purge-injection has shown potential as a tool for breast cancer screening. In the future, efforts will be focused on expanding the sample size for external validation and employing appropriate methods to control the influence of clinical factors, further enhancing the reliability of this method.

## Figures and Tables

**Table 1:** A list of 24 metabolites that appeared in the spectra of QC sample under acid-salted condition.

Measured Mass (Th)	Theoretical Mass (Th)	Mass Error (ppm)	Characteristic Peaks	Chemicals
47.0495	47.0496	−2	C_2_H_6_O·H^+^	ethanol
45.0328	45.0340	−27	C_2_H_4_O·H^+^	acetaldehyde
48.0031	48.0033	−4	CH_4_S^+^	methanethiol
49.0106	49.0112	−12	CH_4_S·H^+^	
57.0338	57.0341	−4	C_3_H_4_O·H^+^	acrolein
59.0498	59.0496	3	C_3_H_6_O·H^+^	acetone
77.0603	77.0602	1	C_3_H_6_O·H_3_O^+^	
117.0915	117.0915	0	(C_3_H_6_O)_2_·H^+^	
61.0280	61.0289	−15	C_2_H_4_O_2_·H^+^	acetic acid
79.0398	79.0395	4	C_2_H_4_O_2_·H_3_O^+^	
73.0652	73.0653	−1	C_4_H_8_O·H^+^	2-butanone
91.0754	91.0759	−5	C_4_H_8_O·H_3_O^+^	
144.1131	144.1150	−13	C_8_H_16_O_2_^+^	octanoic acid
145.1225	145.1228	−2	C_8_H_16_O_2_·H^+^	
83.0715	83.0735	−24	C_5_H_9_N^+^	pentanenitrile
87.0808	87.0810	−2	C_5_H_10_O·H^+^	2-pentanone
105.0915	105.0915	0	C_5_H_10_O·H_3_O^+^	
173.1519	173.1542	−13	(C_5_H_10_O)_2_·H^+^	
88.0346	88.0346	0	C_4_H_8_S^+^	methyl allyl sulfide
92.0629	92.0626	3	C_7_H_8_^+^	toluene
93.0581	93.0578	2	C_6_H_7_N^+^	3-methylpyridine
93.9908	93.9910	−2	C_2_H_6_S_2_^+^	disulfide, dimethyl
96.0576	96.0575	1	C_6_H_8_O^+^	2,5-dimethylfuran
97.0507	97.0527	−21	C_5_H_7_NO^+^	2,5-dimethyloxazole
101.0599	101.0602	−3	C_5_H_9_O_2_^+^	2,3-pentanedione
101.0955	101.0966	−11	C_6_H_12_O·H^+^	2-hexanone
107.0713	107.0735	−21	C_7_H_9_N^+^	2,6-lutidine
110.0725	110.0731	−5	C_7_H_10_O^+^	2-propylfuran
114.0135	114.0139	−4	C_5_H_6_OS^+^	2-methoxythiophene
115.1112	115.1123	−10	C_7_H_14_O·H^+^	2-heptanal
136.1240	136.1252	−9	C_10_H_16_^+^	limonene
139.1120	139.1123	−2	C_9_H_14_O·H^+^	2-pentylfuran

**Table 2:** Identification of differential metabolites in the urine samples between BC patients and healthy controls (CTL).

VOMs	Chemical Formula	Characteristic Peaks	Ratio	*p*-Value	VIP	Ref.
acrolein	C_3_H_4_O	C_3_H_4_O·H^+^	2.00	2.57 × 10^−5^	1.63	[33]
2-butanone	C_4_H_8_O	C_4_H_8_O·H^+^	1.92	8.66 × 10^−5^	1.54	[34,35]
		C_4_H_8_O·H_3_O^+^	1.58	0.0016	1.32	
2-pentanone	C_5_H_10_O	C_5_H_10_O·H^+^	0.53	0.0062	1.17	[34,35]
		(C_5_H_10_O)_2_·H^+^	0.38	2.51 × 10^−4^	1.47	
methyl allyl sulfide	C_4_H_8_S	C_4_H_8_S^+^	0.16	0.0012	1.37	[30,36]
3-methylpyridine	C_6_H_7_N	C_6_H_7_N^+^	2.16	0.0043	1.14	[20]
2-hexanone	C_6_H_12_O	C_6_H_12_O·H^+^	0.56	7.30 × 10^−4^	1.12	[3,37]
2-methoxythiophene	C_5_H_6_OS	C_5_H_6_OS^+^	0.48	0.0097	1.11	[4,30]
2-pentylfuran	C_9_H_14_O	C_9_H_14_O·H^+^	0.46	2.18 × 10^−5^	1.38	[3,37]
octanoic acid	C_8_H_17_O_2_	C_8_H_16_O_2_·H^+^	0.45	0.0108	1.21	[4]

Note: The value of “Ratio” is obtained by dividing the average concentration of BC by the average concentration of CTL.

## Data Availability

All data are contained in the article and Supplementary Materials.

## Supplementary Materials

The following supporting information can be downloaded at: https://www.mdpi.com/article/10.3390/metabo13070870/s1, Figure S1: Schematic diagram of the HPPI-TOFMS.; Figure S2: (a) Principal component analysis (PCA) score plot; (b) RSD distribution for ion features in QC samples. Table S1: The result of ROC analysis for individual metabolites.
