# Supplementary material for: Rapid Detection of Volatile Organic Metabolites in Urine by High-Pressure Photoionization Mass Spectrometry for Breast Cancer Screening: A Pilot Study
Source: Metabolites. 2023 Jul 21;13(7):870. doi: 10.3390/metabo13070870 (PMC10385751; doi:10.3390/metabo13070870)
Supplement: Supplementary file 1 [file metabolites-13-00870-s001.zip › Supporting information.pdf]

Rapid Detection of Volatile Organic Metabolites in Urine by High-pressure Photoionization Mass Spectrometry for Breast Cancer Screening: A Pilot Study

Ming Yang [1,2,3], Jichun Jiang [2,3], Lei Hua [2,3], Dandan Jiang [2,3], Yadong Wang [4], Depeng Li [1], Ruoyu Wang [4], Xiaohui Zhang [1,*] , and Haiyang Li [2,3,*]

1 Key Laboratory of Separation Science for Analytical Chemistry, Dalian Institute of Chemical Physics, Chinese Academy of Sciences, Dalian 116023, China; yangm@dicp.ac.cn (M.Y.); jjc@dicp.ac.cn (J-C.J.); lhua@dicp.ac.cn (L.H.); jiangdandan@dicp.ac.cn (D-D.J.)

2 College of Environment and Chemical Engineering, Dalian University, Dalian 116000, China; lidepeng@dlu.edu.cn(D-P.L.)

3 Center for Advanced Mass Spectrometry, Dalian Institute of Chemical Physics, Chinese Academy of Sciences, Dalian 116023, China;

4 Department of Oncology Medicine, Affiliated Zhongshan Hospital of Dalian University, Dalian 116023, China; 2664126@163.comwangyadong@dlu.edu.cn (Y-D.W.); wangruoyu1963@163.com (R-Y.W.)

* Correspondence: zhangxiaohui@dlu.edu.cn (X-H.Z.); hli@dicp.ac.cn (H.-Y.L.)

S1 RF-only quardrupole TOFMS

The home-built time-of-flight mass spectrometer (TOFMS) shown in Figure S1 consisted of a high-pressure photoionization source, an ion transmission system, and an orthogonal acceleration TOF (oaTOF) mass analyzer. Gas-phase analytes were directly introduced into the ion source through a 250 μm i.d., 50 cm long stainless steel capillary with a sampling flow rate of about 30 mL/min. The ion source was pumped by a 3.3 L/s rotary pump (Edwards Ltd., U.K.), which was also used as the fore pump of a three-stage split-flow turbo molecular pump in the MS instrument. The ion source pressure was adjustable in the range of 10-1000 Pa with a vacuum ball valve, which was fixed between the rotary vane pump and the ion source chamber. The ion transmission system, composed of a radio frequency (RF) -only quadrupole, a Skimmer 2 electrode and a rectangular einzel lens, was designed for efficient ion transmission from the ion source at hPa into the oaTOF mass analyzer at a high vacuum of 10^{-5} Pa. The RF-only quadrupole was constructed of four 12 mm o.d., 130 mm long stainless-steel rods in parallel, which was driven by a home-made RF power supply with the optimized RF frequency at 2 MHz and $V_{p\text{-}p}$ at 300 V. The einzel lens, separated from the RF-only quadrupole by the Skimmer-2 electrode with a 1.5 mm i.d. orifice, was employed to further focus and shape the ion beam. The ion beam was then guided through a 1×10 mm slit into the extraction region of the oaTOF analyzer. The RF-only quadrupole region, electrostatic field region and the mass analyzer regions were differentially pumped by the three-stage split-flow turbo molecular pump (Leybold Vacuum GmbH TW 250/200/40) with 40 L/s, 200L/s and 250 L/s, respectively. The oaTOF mass analyzer used in this instrument was a reflection TOFMS in V-mode. A 33 mm chevron MCP detector with a 50 Ω conical anode was used to collect the ions, and signals were recorded using a 100-ps time-to-digital converter (TDC) (model 9353, Ametec Inc., Oak Ridge, U.S.A) at a repetition rate of 25 kHz. A mass resolving power of about 5000 (FWHM) was achieved with a 0.5 m field-free drift tube.

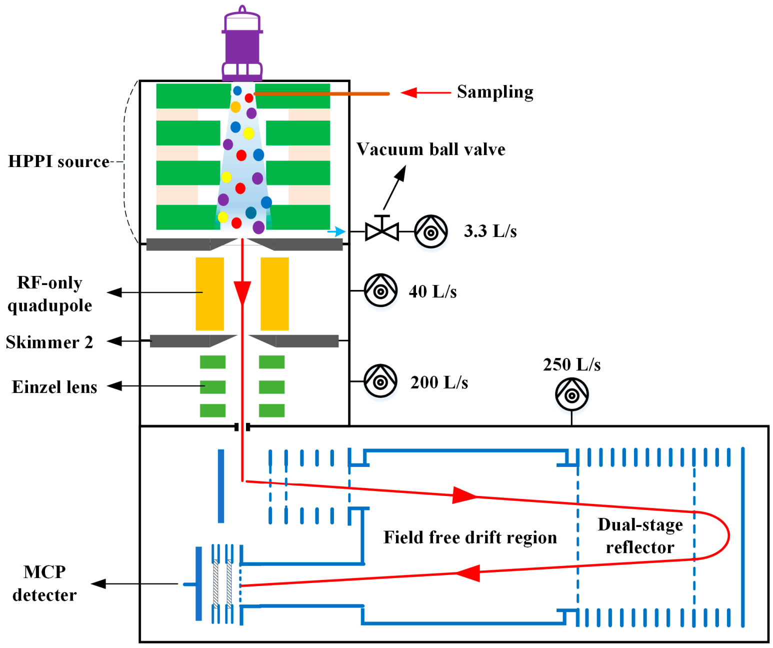

Figure S1 Schematic diagram of the HPPI-TOFMS.

S2 Evaluation of the stability of the experimental method

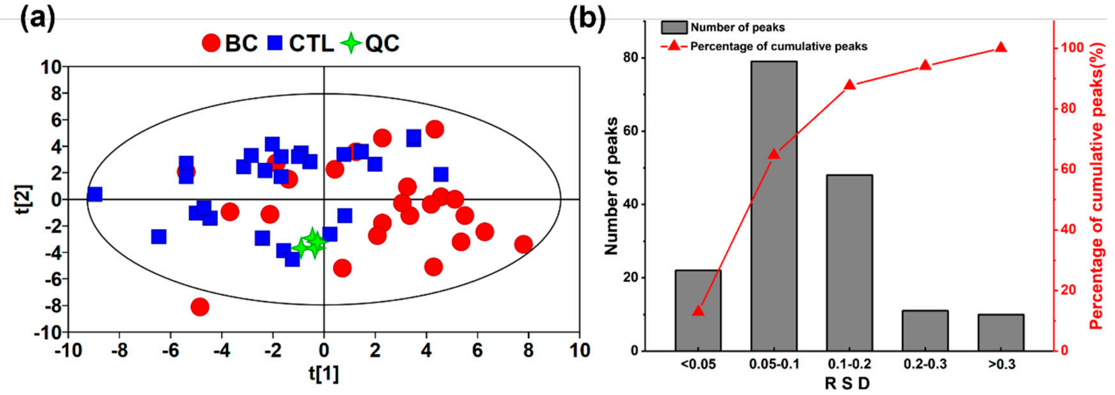

Figure S2 (a) Principal component analysis (PCA) score plot; (b) RSD distribution for ion features in QC samples.

S3 The result of receiver operating characteristic curve for individual metabolites

Table S1 The result of ROC analysis for individual metabolites

Possible VOMs	Characteristic peaks	AUC	Sensitivity	Specificity
acrolein	$C_3H_4O{\cdot}H^+$	0.844	0.8	0.7
2-butanone	$C_4H_8O{\cdot}H^+$	0.821	0.8	0.8
	$C_4H_8O{\cdot}H_3O^+$	0.757	0.6	1
2-pentanone	$C_5H_{10}O{\cdot}H^+$	0.724	0.7	0.7
	$(C_5H_{10}O)_2{\cdot}H^+$	0.799	0.8	0.7
methyl allyl sulfide	$C_4H_8S^+$	0.763	0.7	0.7
3-methylpyridine	$C_6H_7N^+$	0.733	0.6	0.7
2-hexanone	$C_6H_{12}O{\cdot}H^+$	0.776	0.8	0.8
2-methoxythiophene	$C_5H_6OS^+$	0.637	0.7	0.6
2-pentylfuran	$C_9H_{14}O{\cdot}H^+$	0.847	0.8	0.8
octanoic acid	$C_8H_{16}O_2{\cdot}H^+$	0.708	0.6	0.9
